# Tailored extraction and ion mobility-mass spectrometry enables isotopologue analysis of tetrahydrofolate vitamers

**DOI:** 10.1007/s00216-023-04786-5

**Published:** 2023-06-22

**Authors:** Bernd M. Mitic, Diethard Mattanovich, Stephan Hann, Tim Causon

**Affiliations:** 1 University of Natural Resources and Life Sciences Vienna, Department of Chemistry, Insitute of Analytical Chemistry, Muthgasse 18, 1190 Vienna, Austria; 2 University of Natural Resources and Life Sciences, Department of Biotechnology, Institute of Microbiology and Microbial Biotechnology, Vienna, Muthgasse 18, 1190 Vienna Austria

**Keywords:** Tetrahydrofolate pathway, Isotopologue ratio analysis, ^13^C-Labelling, Ion mobility-mass spectrometry, Vitamers

## Abstract

Climate change directs the focus in biotechnology increasingly on one-carbon metabolism for fixation of CO_2_ and CO_2_-derived chemicals (e.g. methanol, formate) to reduce our reliance on both fossil and food-competing carbon sources. The tetrahydrofolate pathway is involved in several one-carbon fixation pathways. To study such pathways, stable isotope-labelled tracer analysis performed with mass spectrometry is state of the art. However, no such method is currently available for tetrahydrofolate vitamers. In the present work, we established a fit-for-purpose extraction method for the methylotrophic yeast *Komagataella phaffii* that allows access to intracellular methyl- and methenyl-tetrahydrofolate (THF) with demonstrated stability over several hours. To determine isotopologue distributions of methyl-THF, LC-QTOFMS provides a selective fragment ion with suitable intensity of at least two isotopologues in all samples, but not for methenyl-THF. However, the addition of ion mobility separation provided a critical selectivity improvement allowing accurate isotopologue distribution analysis of methenyl-THF with LC-IM-TOFMS. Application of these new methods for ^13^C-tracer experiments revealed a decrease from 83 ± 4 to 64 ± 5% in the M + 0 carbon isotopologue fraction in methyl-THF after 1 h of labelling with formate, and to 54 ± 5% with methanol. The M + 0 carbon isotopologue fraction of methenyl-THF was reduced from 83 ± 2 to 78 ± 1% over the same time when using ^13^C-methanol labelling. The labelling results of multiple strains evidenced the involvement of the THF pathway in the oxygen-tolerant reductive glycine pathway, the presence of the in vivo reduction of formate to formaldehyde, and the activity of the spontaneous condensation reaction of formaldehyde with THF in *K. phaffii*.

**Supplementary Information:**

The online version contains supplementary material available at 10.1007/s00216-023-04786-5.

## Introduction

In recent years, one-carbon metabolism of microbes has become an important focus for strategies addressing climate change by biotechnological development. Assimilation of CO_2_ or alternative one-carbon sources, which can be electrochemically derived from CO_2_ (e.g. methanol and formate [1, 2], is of central interest. Several of these one-carbon assimilation pathways proceed via the tetrahydrofolate pathway, for example the serine cycle of *Methylorubrum extorquens* [3, 4]*,* the reductive acetyl-CoA (Wood-Ljungdahl) pathway of acetogenic bacteria and archaea [5, 6], the oxygen-sensitive reductive glycine pathway of *Desulfovibrio desulfuricans* [7], the recently detected oxygen-tolerant reductive glycine pathway in *Komagataella phaffii* [8], and their synthetic integration in model organisms such as *Escherichia coli* and *Saccharomyces cerevisiae* [9–13]. In analytical terms, tracer-based metabolomics studies are the gold standard for investigating these pathways, but interpretation of the tetrahydrofolate pathway currently relies on measurement of downstream metabolites such as amino acids due to the instability of these vitamers in metabolomics workflows [4, 8, 14, 15]. Nevertheless, development of a direct measurement method would allow the deepening of biochemical knowledge of the tetrahydrofolate pathway contributions. In addition, it would deliver essential data for improving pathway activity and efficiency by determining bottlenecks and performing pathway activity-based expression level fine-tuning of tetrahydrofolate pathway enzymes [16].

**Fig. 1:**
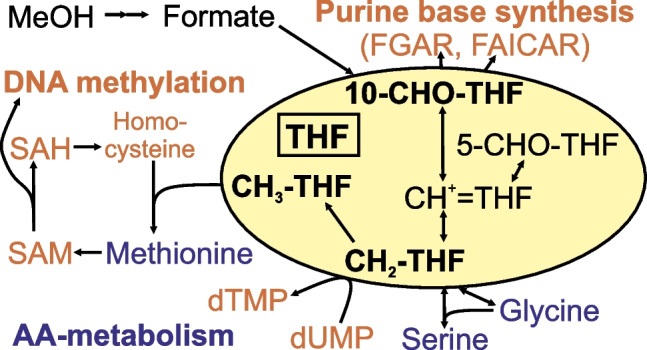
Schematic illustration of the most important tetrahydrofolate pathways for amino acid metabolism and DNA synthesis (methylation, purine, and pyrimidine base synthesis). The tetrahydrofolate pool is represented in the yellow area. THF, tetrahydrofolic acid; SAM, S-adenosylmethionine; SAH, S-adenosylhomocysteine; FGAR, N-formylglycinamide ribonucleotide; FAICAR, 5-formamido-4-imidazolecarboxamide ribonucleotide

Harbouring the vitamin B9 vitamers tetrahydrofolate (**THF**), 5- and 10-formyl-THF (**5-** and **10-CHO-THF**), methenyl-THF (**CH**^**+**^** = THF**), methylene-THF (**CH**_**2**_**-THF**), and methyl-THF (**CH**_**3**_**-THF**), the tetrahydrofolate pathway has broad relevance outside of microbial biotechnology. It is involved in DNA synthesis [17, 18], DNA methylation [19], and amino acid metabolism [20], and serves as a general methyl group donor (Fig. 1). The activity of the tetrahydrofolate pathway and the precursor (folic acid) are essential for fast cell proliferation (e.g. foetal growth during pregnancy and cancer) while deficiencies can cause diseases like neural tube defects, brain abnormalities, or cancer [21, 22]. The focus on human health research has driven wide-ranging analytical method developments for tetrahydrofolate vitamer measurements [23–25], but a key limitation for all of these methods is degradation and interconversion of the target analytes [26–29] rendering quantification of most native forms impossible [30]. The use of buffers and antioxidants such as ascorbic acid and mercaptoethanol can prevent pH- and oxidation-related changes affecting stability, but cannot be applied under all conditions and also does not prevent all possible interconversions [26, 31, 32]. Alternatively, the entire pool can be reduced to CH_3_-THF with sodium borohydride or cyanoborohydride using a deuterated form of the reducing agent to allow the quantification of vitamers [33, 34]. A suitable method for quantifying different tetrahydrofolate pathway intermediates in a stable form is derivatisation with deuterated sodium cyanoborohydride and deuterated ^13^C-labelled formaldehyde [35]. However, this approach cannot be directly adapted to tracer-based pathway analysis in a single fermentation experiment as, unlike for vitamers with natural distributions, the ^13^C-label contribution cannot be corrected from the contribution of ^2^H. In the case of tetrahydrofolate vitamers, an instrument offering a resolving power of > 500 000 operating at high acquisition speeds would be needed to resolve these contributions.

To access the vitamers of interest for analytical measurements without a derivatisation step requires a fit-for-purpose metabolite extraction procedure. In comparison to mammalian cells used in clinical studies, microbial cells, especially yeasts, possess a strong cell wall that requires harsher conditions to access intracellular metabolites. However, the application of high temperature for extraction also promotes metabolite interconversions which falsify analytical results [27]. While existing extraction methods for tetrahydrofolate vitamers use stabilsing agents during the extraction to inhibit interconversion [36-38], the proof that no result-falsifying conversion takes place during the extraction is still missing. More importantly, no ^13^C-tracer-based isotopologue distribution method to assess pathway details and contributions within the tetrahydrofolate pathway has been established to date.

Therefore, we aimed to (1) establish a metabolite extraction procedure free of result-falsifying conversions of tetrahydrofolate vitamers in all procedural steps, (2) avoid laborious and time-consuming sample preparation steps such as solid-phase extraction (SPE) [34, 38–41], and (3) develop a set of multi-dimensional analytical methods allowing the interference-free isotopologue distribution analysis of tetrahydrofolate vitamers. To achieve this, we used ESI–MS in combination with a reversed-phase liquid chromatography (RPLC) separation instead of HILIC [35] to ensure compatibility with aqueous separation conditions necessary for routine use of the sample stabilisation buffer. Following method development with quantitative RPLC-MS/MS, both two-stage high-resolution MS (QTOFMS) and ion mobility high-resolution MS (IM-TOFMS) were harnessed to ensure interference-free isotopologue distribution analysis. Finally, we applied the new extraction and instrumental methods for a ^13^C-tracer experiment in *K. phaffii*, which served as proof of principle and enabled a deeper insight into the role of the tetrahydrofolate pathway in *K. phaffii*’s C1 metabolism.

## Materials and methods

### Chemicals

Tetrahydrofolate pathway intermediate standards, (6S)-5,6,7,8-tetrahydrofolic acid (**THF**); (6S)-5-formyl-5,6,7,8-tetrahydrofolic acid, calcium salt (**5-CHO-THF**); ((6R,S)-5,10-methenyl-5,6,7,8-tetrahydrofolic acid chloride (**CH**^**+**^** = THF**); (6R,S)-5,10-methylene-5,6,7,8-tetrahydrofolic acid, calcium salt (**CH**_**2**_**-THF**); and (6S)-5-methyl-5,6,7,8-tetrahydrofolic acid, calcium salt (**CH**_**3**_**-THF**) were obtained from Schircks Laboratories, Switzerland. The stabilising buffer ingredients used were BioScience-Grade HEPES (Carl Roth GmbH, Germany), 45% (v/v) ammonium hydroxide and sodium ascorbate (Sigma, USA), and 2-mercaptoethanol (VWR, USA). Rat serum (Sigma, USA) was used for de-polyglutamation. MS-grade water (Chromasolv LC–MS, Honeywell, USA) was used for the buffer and extraction and HPLC-grade ethanol (Carl Roth GmbH) for extraction. For HPLC–MS, ultrapure water from a Milli-Q IQ 7000 purification system and LC-Pak® polisher cartridge (Merck Chemicals and Life Science GmbH, Austria), LC–MS-grade acetonitrile (Chromasolv, Honeywell), and LC–MS ultra-grade formic acid (Fluka, Honeywell) were used. The calibration solution used for IM-QTOFMS tuning and ^*DT*^*CCS*_*N2*_ calibration was prepared following the manufacturer’s instructions using ESI-L Tune Mix G1969-85,000 and HP-0321 Agilent Biopolymer Reference Kit from Agilent Technologies, USA.

Enzymes used for cloning were sourced from New England Biolabs GmbH, Germany. For cultivations, YNB and hypoxanthine (Sigma-Aldrich GmbH, USA) were used with the ^nat^C-carbon sources glycerol, methanol, and sodium formate (Carl Roth GmbH). ^13^C-sodium formate was purchased from Sigma-Aldrich GmbH and ^13^C-methanol from CortecNet, France. For pH control in the bioreactor, phosphoric acid and potassium hydroxide (Carl Roth GmbH) were used.

### Yeast strains for method development and labelling experiments

All strains are based on *Komagataella phaffii* (*Pichia pastoris*) CBS7435 [42] and are listed in Table 1. The DasKO strain has the xylulose 5-phosphate pathway (the main methanol assimilation route) blocked. The MisKO strain has the tetrahydrofolate pathway additionally knocked out, rendering the formate fixation route inactive*.* Both strains were recently constructed and used [8]. For the overexpression strain MisOE, the coding sequence of the mitochondrial homolog *MIS1-1* and the cytosolic homolog *ADE3* of *S. cerevisiae* (S288C) was taken from our recent study [8]. Plasmids were constructed by Golden Gate cloning [43]. Promoters, terminators, and plasmid backbones from previous studies [43] were used. For the creation of the overexpression strain, 3 µg of the final overexpression plasmid (BB3eH_pDAS1_MIS1-1_IDP1tt_pDAS2 _ADE3_RPP1Btt) was linearised with *SmaI* and transformed into the DasKO strain as previously described [44].

**Table 1:** Names and genotype of strains used in this study. The WT was described previously [42], the DasKO and MisKO strains were constructed in previous work [8], and the MisOE strain was constructed in the present work

Strain abbreviation	Strain name and genotype
WT	CBS7435 (wild type)
DasKO	CBS7435 *das1Δdas2Δ*
MisKO	CBS7435 *das1Δdas2Δ mis1-1Δmis1-2&3Δ::loxP-kanMX-loxP*
MisOE	CBS7435 *das1Δdas2Δ P*_*DAS1*_*MIS1-1 P*_*DAS2*_*ADE3*

### LC–MS/MS method for relative quantification of tetrahydrofolate vitamers

A method combining liquid chromatography and triple quadrupole mass spectrometry (LC–MS/MS, TSQ Vantage Triple Quadrupole, Thermo Fisher Scientific GmbH, USA) was established for the sensitive relative quantification of tetrahydrofolate vitamers (i.e. without stable isotope-labelled internal standards due to lack of availability for some vitamers). The LC column (Atlantis T3, 3 µm, 2.1 × 150 mm, Waters, USA) was operated at 40 °C with a flow of 300 µL min^−1^ applying a linear gradient program with 0.1% v/v formic acid (A) and acetonitrile (B) within 10 min. The heated electrospray source (HESI) was operated in positive mode and the MS/MS acquired 12 optimised transitions in single-reaction monitoring (SRM) using collision-induced dissociation (CID) with argon as collision gas (Table 2). Detailed ESI–MS settings and the LC gradient are presented in Supplementary Information Tables S1 and S2. LC–MS/MS data evaluation was performed with Tracefinder 5.0 (Thermo Fisher Scientific GmbH). Extracted ion chromatograms of quantifying transitions were integrated and the resulting areas used for data display and evaluation of the stability tests. The qualitative transitions were employed for identity confirmation. LC–MS/MS data was additionally visualised using Skyline.

**Table 2:** LC–MS/MS transitions for analysis of tetrahydrofolate vitamers (see Supplementary Information Table S1 and Table S2 for full method details)

Transition name	Ion species	Precursor (Da)	Product (Da)	SRM collision energy (V)	Type	RT (min)
THF_446.2_299.1	[M + H]^+^	446.2	299.1	18	Quantifying	3.75
THF_446.2_166.1	[M + H]^+^	446.2	166.1	18	Qualifying	3.75
THF_446.2_120.0	[M + H]^+^	446.2	120.0	18	Qualifying	3.75
CH^+^ = THF_456.2_412.2	[M]^**+**^	456.2	412.2	20	Quantifying	3.90
CH^+^ = THF_456.2_327.2	[M]^+^	456.2	327.2	25	Qualifying	3.90
CH^+^ = THF_456.2_282.2	[M]^+^	456.2	282.2	30	Qualifying	3.90
CH_2_-THF_458.2_311.1	[M + H]^+^	458.2	311.1	18	Quantifying	4.21
CH_2_-THF_458.2_166.1	[M + H]^+^	458.2	166.1	18	Qualifying	4.21
CH_3_-THF_460.2_313.1	[M + H]^**+**^	460.2	313.1	18	Quantifying	3.84
CH_3_-THF_460.2_180.1	[M + H]^+^	460.2	180.1	18	Qualifying	3.84
5-CHO-THF_474.2_327.1	[M + H]^**+**^	474.2	327.1	18	Quantifying	4.22
5-CHO-THF_474.2_299.1	[M + H]^+^	474.2	299.1	18	Qualifying	4.22

### Assessment of tetrahydrofolate vitamer stability during storage on the autosampler and during metabolite extraction procedures

To assess the stability of the tetrahydrofolate vitamers, single stocks of each compound in concentrations of 0.1 µmol L^−1^ and 1 µmol L^−1^ were prepared in the initial LC eluent composition (95:5 0.1% v/v formic acid:acetonitrile) and in an established HEPES buffer (50 mmol L^−1^ HEPES buffer in MS-grade H_2_O with 1% (v/v) 2-mercaptoethanol and 1% (w/v) sodium ascorbate adjusted to pH 8 with concentrated ammonium hydroxide (25%)) [32]. Using the LC–MS/MS method, the single standards were injected within 10 min of preparation and subsequently analysed every 8 h up to a total of 48 h. In addition, eluent and HEPES buffers were injected before and after each single stock injection to assess carryover between injections.

To investigate interconversions of 5-CHO-THF, CH^+^ = THF, and CH_3_-THF occurring during extraction, 10 µL of 100 µmol L^−1^ stock solutions was added to empty sample tubes (yielding 1 µmol L^−1^ after reconstitution in water) and LC–MS/MS results from these extracted standard solutions compared to the corresponding single stock (which were not subject to the extraction procedure). After data evaluation, peak areas were normalised to the corresponding area from a measurement of single stocks, as only interconversion (and not degradation) was of interest in this experiment.

### Metabolic sampling and quenching

For each sample, a volume of cell broth corresponding to a defined amount of dry cell weight was quenched in a fourfold volume of − 30 °C quenching solution. The quenching solution was prepared to a final concentration of 125 mmol L^−1^ HEPES and 2.5% (w/v) ascorbic acid with dilution in methanol to yield a 40:60 (v/v) aqueous:methanol composition. Prior to final dilution in methanol, the aqueous mixture was adjusted to pH 8 with 25% (v/v) ammonia. The cold mixture was vortexed for 4 s and immediately transferred onto a cold and quenching solution-moistened cellulose acetate filter (0.45 µm, Sartorius Stedim Biotech GmbH, Germany). Filtered cells were washed with 10 mL of quenching solution. The filter was stored in 15-mL tubes at − 80 °C for a maximum of 30 h until extraction.

### Tetrahydrofolate vitamer extraction and sample preparation procedure

The aqueous part of the extraction buffer was identical to the stabilisation buffer containing 50 mmol L^−1^ HEPES buffer in MS-grade H_2_O adjusted to pH 8 with concentrated ammonium hydroxide with 1% (v/v) 2-mercaptoethanol and 1% (w/v) sodium ascorbate. This buffer was diluted with ethanol to achieve a 75% v/v ethanol-containing extraction solution. The new extraction procedure is derived from our established boiling ethanol extraction for *K. phaffii* [45, 46]. The extraction solution aliquots (4 mL) were heated to 85 °C in a water bath before extraction. Tubes containing samples (either a cellulose acetate filter with quenched cells or a single-stock standard) were stored on dry ice during extraction. A hot 4-mL aliquot was added to each sample, which was then vortexed immediately for 20 s and placed in an 85 °C water bath for 1.5 min. After that, samples were vortexed again for 10 s before being returned to the bath for further 1.5 min. Tubes were then rapidly cooled on dry ice for 3 min and stored in a cooling box (− 30 °C) until all samples were extracted. Extractions were performed with a carefully managed time plan to ensure that each sample had the same history prior to storage. All tubes were centrifuged at 4000 g for 10 min at − 20 °C. Finally, 2.5 mL of the extract was decanted and dried completely under vacuum at room temperature. Dried extracts were stored at − 80 °C for a maximum of 3 days. Storage stability tests of tetrahydrofolate vitamers at − 80 °C were conducted previously [26]. Dry extracts were resuspended in 625 µL of MS-grade water, resulting in the initial stabilisation buffer concentrations in the reconstituted sample. For the de-polyglutamylation of folates, 32 µL of rat serum was added and the sample incubated at 37 °C with shaking at 180 rpm (Infors HT Multitron, Switzerland) for 3 h. After incubation, the mixture was rapidly cooled on ice and filtered through a 10-kDa spin filter (Merck Millipore, Amicon) at 4 °C. The filtrate was transferred to a dark glass vial with a 100 µL insert and stored in the autosampler at 4 °C until measurement. Biological samples were stored for a maximum of 24 h on the autosampler (4 °C) prior to analysis with LC–MS/MS or LC-IM-QTOFMS.

### LC-IM-QTOFMS for isotopologue distribution analysis

For isotopologue distribution analysis, LC in combination with a drift tube ion mobility-quadrupole-time of flight MS system (LC-IM-QTOFMS; Agilent 1290 Infinity II UPLC, Agilent 6560) was used. The same LC column and conditions as for the LC–MS/MS method were applied. The injection volume was 15 µL. The ion source (Dual Jetstream ESI) was operated in positive mode. Detailed instrument settings are reported in Supplementary Information Tables S3 and S4. All data were acquired with online mass calibration (purine and hexakis(2,2,3,3-tetrafluoropropoxy) phosphazene) in profile mode and converted to centroid after measurement for further data evaluation. Data acquisition was performed with 1000 ms spectrum^−1^ (MS1, TOF), while the isolation width was set to medium (~ 4 m/z) for targeted (MS2, QTOF) acquisition at 7 spectra s^−1^ using a collision energy of 18 V with nitrogen as collision gas for CID.

For IM-TOFMS measurements, 4-bit multiplexing was employed with 3.2-ms trap filling time and 150-µs trap release time. The acquisition rate was 19 IM transients frame^−1^, using a maximum drift time of 50 ms leading to 1 IM frame s^−1^ and 600 TOF transients per IM transient. Prior to sample measurement, a mix of ESI-L Tune Mix and 0.1 mmol/L HP-0321 was used for mass calibration and CCS calibration [47]. To accommodate the reduced dynamic range of IM-QTOFMS in comparison to the LC–MS/MS method, the applied LC gradient was optimised (Supplementary Information Table S3), and two method time segments were employed. The new LC gradient allowed improved signal to noise ratio in the most complex region of the data, while diverting the LC effluent to waste for the first 4 min after injection prevented buffer stabilisation agents saturating the TOFMS detector.

### Isotopologue distribution data analysis

For evaluation of LC-QTOFMS data in Agilent MassHunter Workstation Quantitative Analysis (11.1), extracted ion chromatograms (EICs) corresponding to the accurate masses of CH_3_-THF fragment isotopologues (Supplementary Information Table S5) were evaluated. LC-IM-TOFMS data were recalibrated with Agilent IM-MS Reprocessor and demultiplexed with the PNNL-PreProcessor 4.0 [48]. For evaluation of isotopologue distributions, LC-IM-TOFMS data was filtered by isolating around the target arrival time (drift time) of the corresponding standards in Agilent IM-MS Browser (10.0) and converted to LC–MS format for evaluation in the Quantitative Analysis software (see Supplementary Information Table S6).

Isotopologue fractions were calculated for the detected isotopologues according to Eq. (1):

1 $${Isotopologue\; fraction }_{i}=\frac{{A}_{i}}{{\sum }_{i=0}^{n}{A}_{i}}$$

where *n* is the number of carbon atoms in the metabolite and *A*_*i*_ is the EIC peak area or height of isotopologue.

Evaluation of the centroided data entailed assessment of chromatographic peak area for QTOFMS data, and peak height for IM-TOFMS (for details of assessment, see Supplementary Information S1.3.1). All data were corrected to their full carbon backbone isotopologue distribution using the ICT correction toolbox v.0.04 [49] to allow comparability of precursor and fragment-level carbon isotopologue distribution data.

### Time-resolved bioreactor labelling experiments

The time-resolved labelling experiments were conducted in four bioreactors (1.4-L DASGIP reactors, Eppendorf) in parallel. One reactor containing the WT strain was labelled with ^13^C-sodium formate (99atom% ^13^C) and a second was fed with ^nat^C-sodium formate as a control. Two reactors containing the DasKO strain were used for methanol: one fed with ^13^C-methanol (99atom% ^13^C) and one with ^nat^C-methanol. For inoculation of 100 mL preculture in YPD medium (25 °C, 180 rpm, 21 h), 0.5 mL of a working cell bank (OD_600_ of 8) was used. Bioreactors were filled with 275 mL YNB medium (with 10 g L^−1^ (NH_4_)_2_SO_4_, 0.1 mol L^−1^ potassium phosphate buffer, pH 6) containing 9.7 g L^−1^ glycerol for the batch phase and inoculated with the media exchanged preculture to an OD_600_ of 1. The temperature was controlled at 25 °C for the entire cultivation and pH was maintained at 6.0 by the bioreactor control system using 5 mol L^−1^ H_3_PO_4_ and 5 mol L^−1^ KOH. During the batch phase, the dissolved oxygen concentration was maintained at 30% saturation (DO cascade: stirrer 200–900 rpm, airflow 6–40 sL h^−1^). Within 45 min of the batch end (glycerol depletion), induction was carried out by pulsing either 3 mol L^−1^ formate (WT) to a final concentration of 30 mmol L^−1^ in the reactor or pure methanol (DasKO strain) to 1% (v/v) in the reactor. During the induction phase, the stirrer speed was set to 500 rpm, and the gas flow to 35 sL h^−1^ with 5% CO_2_ and 19.95% O_2_ The initial concentration of formate was adjusted after 4.5 h, and that of methanol after 9.5 h and 19 h, respectively. Metabolic samples were taken in triplicates from each reactor after 1, 4, and 8 h of induction for formate (WT) and after 1, 6, and 24 h of induction for methanol (DasKO strain). For metabolic sampling, 32 mL was taken out of the bioreactor quickly and 10 mL for each replicate was quenched in parallel, corresponding to 50 mg dry cell weight on each filter. More detailed information for this cultivation can be found in Supplementary Information S3. These samples were measured with LC-IM-QTOFMS.

### Shake flask labelling experiment

For the shake flask labelling experiments, precultures were carried out in the same way as for the bioreactor labelling experiment. For the MisKO strain, pre and batch cultures were performed on YPD with 5 mmol L^−1^ hypoxanthine and 100 mg L^−1^ nourseothricin at 30 °C, 180 rpm and the batch culture in 2-L shake flasks for 22–23 h. With the exception of the MisKO strain, the batch cultures were conducted on YNB with 18 g L^−1^ glycerol in 1-L shake flasks for 22–23 h at 25 °C, 180 rpm. For the MisOE strain in the pre culture and batch culture, 200 mg L^−1^ hygromycin was added. The YNB batch cultures were induced 2 h prior to the media exchange to either 1% (v/v) ^nat^C-methanol or 30 mmol L^−1 nat^C-formate with the same carbon source as the corresponding labelling experiment was conducted. During media exchange, cells were centrifuged for 5 min at 4 °C and 1500 g, washed twice with YNB and resuspended in YNB with 1% (v/v) ^13^C-methanol or 30 mmol L^−1 13^C-sodium formate. Labelling experiments were conducted in biological triplicates with 15 mL culture volume at OD_600_ of 25 in 100-mL shake flasks at 25 °C and 180 rpm. For each labelling experiment with a ^13^C-carbon source performed in triplicate, a corresponding ^nat^C-source triplicate was cultivated in parallel as a control. Samples were taken after 6 h of labelling for methanol and 4 h for formate, corresponding to the second sampling point of the reactor experiment.

## Results and discussion

### Stability of tetrahydrofolate vitamers in metabolomics workflow

Many soft extraction methods for tetrahydrofolate vitamers not requiring extreme heat are available for cells which are easy to break up such as mammalian cells or tissues [29, 31, 34], while other methods have been established for organisms with cell walls (e.g. plants, bacteria, and yeasts) that aim to prevent interconversions or degradations by using buffers and antioxidants [14, 26, 34, 36–38, 50]. For yeasts, intracellular tetrahydrofolate vitamers in *S. cerevisiae* can be extracted without derivatisation using stabilising buffers (0.1 mol L^−1^ phosphate buffer at pH 6.1 with 2% sodium ascorbate (w/v) and 0.1% 2-mercaptoethanol (v/v), boiling for 10 min) allowing extraction and measurement of CH_3_-THF and 5-CHO-THF from 50 mg dry yeast with HPLC–UV/FD [37]. Lu et al. extracted unquenched cells with 80:20 methanol:water with 0.1% ascorbic acid, 20 mM ammonium acetate, and 10-min sonication and could measure CH_3_-THF and 5-CHO-THF, but not CH^+^ = THF with LC–MS/MS [36]. Gmelch et al. [38] used a 100 mmol L^−1^ 4-morpholineethanesulfonic acid (MES) hydrate buffer with 10 g L^−1^ ascorbic acid and 1 g L^−1^ dithiothreitol (DTT) adjusted to pH 5.0 with 5 mol L^−1^ NaOH for extraction, boiled for 10 min and quantitatively measured CH_3_-THF, THF, CH^+^ = THF, 5-CHO-THF, and 10-CHO-THF without derivatisation. However, most published analytical methods have not assessed an essential requirement for isotopologue distribution analysis, i.e. the conversion of tetrahydrofolate vitamers during the extraction process. An exception to this was Brouwer et al. [26], who studied pH and temperature stability of folate vitamers using LC–MS/MS. A stabilisation buffer composition established in [32] containing ascorbic acid and mercaptoethanol in HEPES was used across a range of studies involving measurement of tetrahydrofolate vitamers and offered clear potential for application in the present work. As an established boiling ethanol extraction procedure is available for *K. phaffii* metabolomics [45, 46], we aimed to implement this buffer in the standard procedure according to Gonzalez et al., who previously combined a different HEPES buffer composition with boiling ethanol extraction for other metabolites [51].

To this end, the transferability of the stabilisation buffer from [32] needed to be assessed for our application prior to method development. LC–MS/MS measurements (Supplementary Information Fig. S2) revealed that commercially available THF, CH_2_-THF, 5-CHO-THF, CH^+^ = THF, and CH_3_-THF standards were indeed not stable over typical measurement timescales when prepared at concentrations of 0.1 or 1 µmol L^−1^ in 95% water with 0.1% FA and 5% (v/v) acetonitrile. In contrast, THF, 5-CHO-THF, CH^+^ = THF, and CH_3_-THF were observed to be stable when stored at 4 °C for up to 48 h in the stabilisation buffer with issues observed only for CH_2_-THF, which completely converted to THF within 24 h. Of the tetrahydrofolate vitamers, it is noted that 10-CHO-THF is extremely unstable and results will be conflated with those of CH^+^ = THF under the acidic eluent conditions necessary for this study [26]. From the tetrahydrofolate vitamers, it is noteworthy that THF does not harbour the target carbon group of interest for studying one-carbon metabolic fluxes (see Fig. 4a) and, as it is a conversion product of CH_2_-THF, its observed isotopologue pattern would be influenced by both conversion pathways and is therefore not interpretable. This finding correlates with previous experiments [26, 27, 29, 32, 34] and underlines the fact that quantitative measurements of CH_2_-THF and THF without derivatisation cannot be considered reliable [30, 31, 36]. Finally, carryover assessment using buffer blanks injected directly after analysis of the 1 µmol L^−1^ standards showed intensities of 3 orders of magnitude less when compared to the standard confirming that carryover was not an issue with the new LC–MS/MS method.

**Fig. 2:**
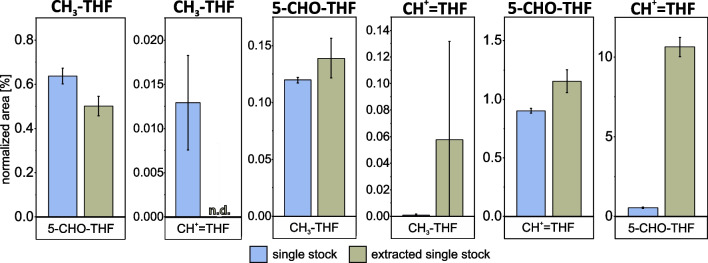
Tetrahydrofolate vitamer stability in new extraction procedure. Single stock standards of each vitamer indicated above the plots were split, one fraction stored at 4 °C (blue) while the second was subject to the entire extraction procedure (olive). The relative abundance comparison of the conversion products or impurities indicated in the box beneath the plot is normalised to the main single stock compound (indicated above) as 100%. Extractions and measurements were performed in triplicate, averages are displayed with ± 1 s error bars

As the stabilisation buffer could prevent oxidation and interconversion of key tetrahydrofolate vitamers, we exchanged the aqueous phase of the boiling ethanol extraction procedure for *K. phaffii* [45, 46] with buffer in order to stabilise the target molecules during the extraction procedure. To determine whether addition of this buffer to the extraction solution was effective, solutions of 5-CHO-THF, CH^+^ = THF, and CH_3_-THF were subject to the entire procedure and results compared to those of individual standards stored at 4 °C. As an indication for interconversion during the extraction, a relative increase in signal intensity of the vitamer is expected when going through the extraction procedure (‘extracted single stock’) in comparison to a stock maintained at 4 °C (‘single stock’). It is noted that all three vitamers are detected in each single stock due to impurities in the standard. The results (Fig. 2) reveal that no appreciable conversion of CH_3_-THF to 5-CHO-THF or CH^+^ = THF, nor of 5-CHO-THF and CH^+^ = THF to CH_3_-THF, was observed. However, approximately 0.1% of 5-CHO-THF converts to CH^+^ = THF and approximately 10% of CH^+^ = THF converts to 5-CHO-THF. Therefore, the conversion of 5-CHO-THF to CH^+^ = THF will only impact the interpretation of CH^+^ = THF isotopologue results if the abundance of 5-CHO-THF is higher than that of CH^+^ = THF. However, the results for 5-CHO-THF can only be interpreted correctly if the intensities of 5-CHO-THF are at least a factor of approximately 10^3^ higher than CH^+^ = THF.

To correctly assess relative intracellular concentrations of the vitamers present in real samples, testing for suitable amounts of biomass of *K. phaffii* and reconstitution volumes of dried extracts was undertaken. Quantities of 10, 25, and 50 mg biomass (cell dry weight) were quenched, extracted with the new procedure, and then dried and reconstituted in 313 or 625 µL H_2_O. The quenching procedure used was adapted from an established HEPES quenching procedure [52], by adding ascorbic acid already to the quenching procedure. Samples were taken at an OD_600_ of 33 from a 200 mL YNB glycerol batch of the DasKO strain in a 2-L shake flask as described in the method section. Results and experimental details can be seen in Supplementary Information Chapter S2.3, Fig. S3 and S4. This revealed that 5-CHO-THF, CH_3_-THF, and CH^+^ = THF were detected in all samples confirming that all target metabolites could be accessed with the new method. Moreover, the intensity of 5-CHO-THF was factor 5 lower than that of CH^+^ = THF meaning that CH^+^ = THF results can be considered valid and not falsified by conversion from 5-CHO-THF. Conversely, results for 5-CHO-THF are not reliable as a substantial fraction is known to derive from intracellular CH^+^ = THF. However, this limitation does not impede pathway interpretation as 5-CHO-THF is a storage molecule and not directly involved in the one-carbon transferring tetrahydrofolate pathway [34]. In summary, CH^+^ = THF and CH_3_-THF, which are of primary importance for the one-carbon pathway, can be measured in *K. phaffii*, evaluated and interpreted without the influence of conversion using the newly developed method.

For subsequent method transfer to a full-scan HRMS instrument, the low signal concentration of vitamers observed in real samples can be a significant limitation. Therefore, samples with high concentrations of vitamers were chosen for method development with IM-QTOFMS. Interestingly, CH^+^ = THF intensities were found to be mainly influenced by the biomass amount and not the reconstitution volume, while the intensities of CH_3_-THF more than doubled when reconstituted in twice the volume. The increased reconstitution volume results in a lower stabilising buffer concentration, which consequently leads to a reduction in ion suppression effects affecting CH_3_-THF, in good agreement with previous results [53]. Finally, the best results for both target analytes were achieved with a combination of 50 mg cell dry weight of *K. phaffii* cells and a reconstitution volume of 625 µL (see Supplementary Information Fig. S4).

### Isotopologue distribution analysis method development

Interference-free analysis of all evaluated isotopologues is a prerequisite for isotopologue distribution analysis. The second criteria to accept a method for this kind of analysis was a profile area of at least 500 for the isotopologue M + 1, in order to satisfy ion counting statistics considerations [54]. We use time of flight TOFMS for isotopologue distribution analysis [8, 55–57] as it provides sufficient resolution for discriminating key isotopologues from isobaric interferences and allows identity confirmation by accurate mass. For the translation of the MS/MS method to the TOFMS platform, we aimed to increase the method ruggedness in terms of matrix effects by improving the separation of the buffer components from the target analytes, increase the signal intensities of key vitamer isotopologues, and avoid unnecessary TOFMS detector saturation and contamination of the ion source. To achieve these goals, the LC gradient time was extended, the sample injection volume was increased to 15 µL, and the first 4 min of eluate was diverted to waste instead of to the ion source.

**Fig. 3:**
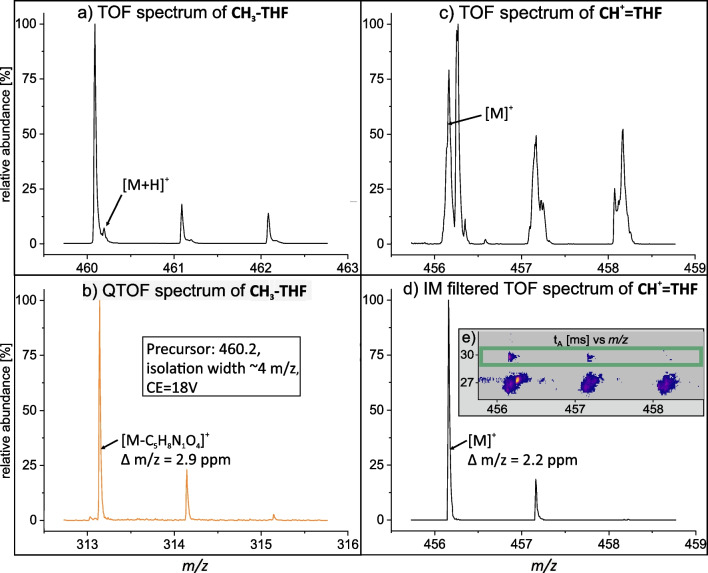
Key LC-IM-QTOFMS results for isotopologue distribution analysis of folate vitamers. CH_3_-THF mass spectra extracted between 4.65 and 4.90 min: (**a**) incompletely resolved TOFMS and (**b**) clean QTOFMS fragments. CH.^+^ = THF mass spectra extracted between 4.68 and 4.95 min: (**c**) incompletely resolved TOFMS and (**d**) clean IM-TOFMS result achieved using (**e**) arrival time filtering (green box)

Despite the optimisation steps performed, both CH^+^ = THF and CH_3_-THF were observed to have major spectral interferences in TOF (MS1) spectra from samples (Fig. 3a and c). Therefore, selective fragmentation of the main precursor ion of the LC–MS/MS method was applied in QTOF (MS2) acquisition mode using the ‘medium’ isolation width (~ 4 m*/z* units) of the quadrupole to ensure concomitant transmission and fragmentation of key isotopologues (M + 0 up to M + 3). As seen in Fig. 3b, the fragment isotopologues of CH_3_-THF were well-resolved from interferences and with sufficient intensities for isotopologue distribution analysis. However, the intensity of the second fragment isotopologue (M + 1) of CH^+^ = THF was generally too weak for reliable interpretations according to ion counting statistics except in samples from the MisKO strain. Therefore, to assess the isotopologue distribution of CH^+^ = THF in all samples on a uniform basis, we instead applied ion mobility (IM) as an additional separation dimension for the MS1 (TOF) level. Additionally, as the observed intensities of target analytes were low, we used IM multiplexing to compensate for the reduced ion utilisation efficiency and potential losses in metabolite transmission [58]. Finally, IM enabled effective resolution of CH^+^ = THF from the interference leading to an interference-free spectrum with sufficient intensities for isotopologue distribution analysis (Fig. 3d).

This successful result is due to the complementary nature of the IM separation compared to LC separation as it involves transport of gas-phase ions of the vitamers in the presence of nitrogen drift gas. For example, while CH^+^ = THF and CH_3_-THF are not baseline separated with LC, they are easily baseline separated in IM due to a large difference of 11.8% in the ^*DT*^*CCS*_*N2*_ values of the vitamer precursor ions (221.5 Å^2^ for CH^+^ = THF, 198.1 Å^2^ for CH_3_-THF) arising from key structural differences (i.e. the closure of the 5-membered ring in CH^+^ = THF and change in charge state location) allowing interference-free assessment of their *m/z* isotopologue distributions (see Supplementary Information Fig. S5). While IM was found to be an ideal solution in our study, we note that the successful analysis of CH^+^ = THF might also be addressed via development and application of additional preconcentration or clean-up steps in combination with LC-HRMS.

**Fig. 4:**
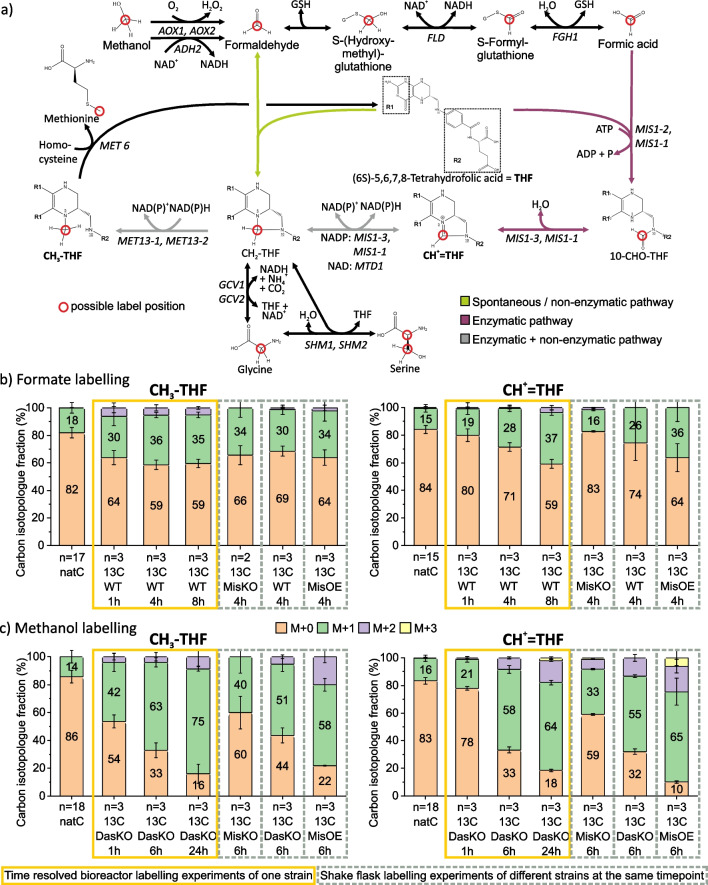
Tetrahydrofolate pathway and isotopologue distribution analysis results from ^13^C labelling experiments. **a** Tetrahydrofolate pathway in *K. phaffii* with gene annotation. **b** Carbon isotopologue distribution analysis results of ^13^C-formate labelling. **c** Carbon isotopologue distribution analysis results of.^13^C-methanol labelling. Yellow boxes (**b**, **c**) highlight the time-resolved bioreactor labelling experiment results of one strain; grey dashed boxes (b, **c**) highlight shake flask labelling experiments of different strains at the same timepoint. All strain names and genotype are found in Table 1. Note: 5-CHO-THF is not shown here as it is a storage molecule not involved in the transfer of the C1 pathway (see Fig. 1)

The relative standard deviation of isotopologue fractions obtained from repeated sampling (bioreactor experiments, Fig. 4b and c) was lower than that of the biological replicates (shake flasks, Fig. 4b and c), which indicates that the uncertainty of measurement of the analytical method is negligible compared to the uncertainty contribution from the biological experiments. This demonstrates that the analytical method is fit-for-purpose, and reliable interpretations of vitamer isotopologue distributions can be made.

### Application of workflow for ^13^C-tracer-based pathway analysis

The new workflow was applied to gain insight into the one-carbon metabolism of *K. phaffii* within the tetrahydrofolate pathway. To this end, several labelling experiments were performed with ^13^C-methanol and ^13^C-formate. For ^13^C-tracer-based pathway assessment, it is expected within a labelling experiment that a metabolite upstream in a pathway contains more of the isotopic label than the downstream metabolites. Therefore, metabolites exhibiting the highest degree or fastest rate of ^13^C label incorporation are an indication for the start of a pathway. The respective isotopologue in each case is denoted by ‘M + x’ indicating the number of ^13^C atoms in the metabolite. A decrease of the M + 0 isotopologue fraction corresponds to an increase of ^13^C content. The carbon isotopologue distribution results and the tetrahydrofolate pathway are shown in Fig. 4.

The importance of the new method for proving the direct involvement of the tetrahydrofolate pathway in the one-carbon metabolism of *K. phaffii*´s methanol and formate assimilation is apparent according to the increase of the ^13^C content of CH^+^ = THF and CH_3_-THF over time (Fig. 4b and c). As we observed a higher degree of label incorporation in CH^+^ = THF and CH_3_-THF in comparison to the three downstream amino acids methionine, serine, and glycine (see Fig. 4a) evaluated in previous work [8], we can further deduce that formate and methanol are assimilated via the tetrahydrofolate pathway within the oxygen-tolerant reductive glycine pathway in this organism.

Both CH^+^ = THF and CH_3_-THF were found to have more label incorporated when cultivated with methanol instead of formate, which is in good correspondence with previous GC-TOFMS data [8]. A reason for this is the higher molar concentration of methanol than formate, and the fact that methanol assimilation and oxidation to intracellular formaldehyde and formate are evolutionary-optimised, i.e. while methanol is a common substrate for *K. phaffii*, it did not evolve to assimilate formate.

In the enzymatic tetrahydrofolate pathway, formate is condensed by the Mis enzymes to 10-CHO-THF, and reacts to CH^+^ = THF, further to CH_2_-THF and finally to CH_3_-THF via the Met enzymes (Fig. 4a). Therefore, CH^+^ = THF is upstream of CH_3_-THF in the enzymatic pathway, but CH_3_-THF was found to be labelled to a higher degree than CH^+^ = THF irrespective of strain, carbon source, or timepoint. The only explanation for this result is that the spontaneous and reversible condensation reaction of formaldehyde with THF to CH_2_-THF is active. The presence of this spontaneous reaction is indicated by the time-resolved labelling experiment and confirmed with the shake flask labelling experiment of the MisKO strain. In this strain, the enzymatic tetrahydrofolate pathway proceeding via 10-CHO-THF is deleted, but CH_3_-THF remains ^13^C-labelled when fed with methanol and formate while CH^+^ = THF also remains ^13^C-labelled when fed with methanol. In the non-enzymatic pathway, methanol and formate are converted to formaldehyde by oxidation and reduction, respectively. Formaldehyde reacts spontaneously with THF to form CH_2_-THF, further via the Mtd1 enzyme in the MisKO strain to CH^+^ = THF and to CH_3_-THF via the Met enzymes. While the WT and DasKO strains harbour the native activity of the enzymatic pathway, it is deleted in the MisKO strain and enhanced in the MisOE strain. Based on the analytical results, the activity of the enzymatic pathway to CH_2_-THF is evidenced by the increased labelling observed in the tetrahydrofolate vitamers within the WT and the DasKO strains in comparison to the MisKO strain. Moreover, the MisOE strain exhibits even greater ^13^C incorporation compared to the WT and DasKO strains further supporting the hypothesis that, in addition to the spontaneous (non-enzymatic) pathway, the enzymatic pathway is also active.

As the activity of both tetrahydrofolate pathways for fixation of methanol has been previously shown for the serine cycle in *M. extorquens* [4] and in an engineered *E. coli* strain [14] (for which the enzymatic pathway mainly contributes to growth), finding experimental evidence of the spontaneous reaction in *K. phaffii* was not unexpected. However, recent GC-TOFMS analysis of amino acids (Gly, Ser) evidenced only the enzymatic tetrahydrofolate pathway, but the label incorporation in the MisKO strain coming directly from formate or methanol could not be confirmed [8]. The new method covers this gap by allowing us to observe and confirm the condensation reaction of formaldehyde and THF that required an interference-free mass spectrum of CH +  = THF achieved using additional IM separation without further modification to the primary LC–MS method. Taken together, these results demonstrate the value of the new method for tetrahydrofolate pathway analysis and the importance of relying not only on the analysis of downstream metabolites for deciphering tetrahydrofolate pathway activities and for isotopologue distribution analysis that goes beyond the scope of the application presented here.

## Conclusion

We established a new modular workflow from cell quenching to analytical measurements of CH_3_-THF and CH^+^ = THF for isotopologue distribution analysis, which was previously lacking for tetrahydrofolate vitamers. The incorporation of a HEPES buffer combined with ascorbic acid and β-mercaptoethanol in the extraction solution was key for stabilisation of the target tetrahydrofolate vitamers during the new extraction procedure. For ensuring accurate isotopologue distribution analysis, a combination of high-resolution mass spectrometry, CID fragment spectra and additional IM separation provided the required selectivity for assessment of CH_3_-THF and CH^+^ = THF in *K. phaffii* metabolite extracts, respectively. The new method allowed an isotopologue distribution analysis to be performed without further laborious preconcentration steps or derivatisation. In particular, the novel use of IM separation for isotopologue distribution analysis demonstrates broader potential for pathway analysis where key molecular information can be revealed with only minor modifications to existing LC-HRMS methods and data processing workflows.

Application of the new workflow allowed an outstanding finding to be revealed; namely that the spontaneous and non-enzymatic condensation reaction of formaldehyde and tetrahydrofolate is present and active in vivo in *K. phaffii* in addition to the enzymatic tetrahydrofolate pathway. Additionally, the reduction of formate to formaldehyde when cultivated on formate was observed, which is a novel feature of the one-carbon metabolism of *K. phaffii*.

Finally, while applied to yeast biotechnology in the present work, the new modular workflow should be applicable to studies involving other microbial strains to investigate their tetrahydrofolate pathway, understand their one-carbon metabolism in more detail, and use the results to further optimise their metabolic activity and efficiency.

## Supplementary Information

Below is the link to the electronic supplementary material.

Supplementary file1 (PDF 797 kb)
