# Supplementary material for: Tailored extraction and ion mobility-mass spectrometry enables isotopologue analysis of tetrahydrofolate vitamers
Source: Anal Bioanal Chem. 2023 Jun 22;415(21):5151–63. doi: 10.1007/s00216-023-04786-5 (PMC10404201; doi:10.1007/s00216-023-04786-5)
Supplement: Supplementary file 1 — Supplementary file1 (PDF 797 kb) [file 216_2023_4786_MOESM1_ESM.pdf]

Supplementary Information for:

Tailored extraction and ion mobility-mass spectrometry enables isotopologue analysis of tetrahydrofolate vitamers

Bernd M. Mitic[1,2], Diethard Mattanovich[2], Stephan Hann[1], Tim Causon[1,*]

[1)] University of Natural Resources and Life Sciences, Vienna, Department of Chemistry, Institute of Analytical Chemistry, Muthgasse 18, 1190 Vienna, Austria

[2)] University of Natural Resources and Life Sciences, Vienna, Department of Biotechnology, Institute of Microbiology and Microbial Biotechnology, Muthgasse 18, 1190 Vienna, Austria

* Corresponding author: tim.causon@boku.ac.at

Table of Contents

Overview of Tables 2
Overview of Figures 2
S1 Detailed analytical method information 3
S1.1. LC-MS/MS 3
S1.2. LC-IM-QTOFMS 3
S1.3. Isotopologue distribution data analysis 4
S1.3.1 Assessment of data evaluation workflows 5
S2 Analytical developments for tetrahydrofolate vitamers 5
S2.1. Fragment structure assignment 5
S2.2. Tetrahydrofolate vitamer stability and conversion analysis 6
S2.3. Extraction and measurement of tetrahydrofolate vitamers of *K. phaffii* 7
S2.3. Isotopologue distribution analysis method development 9
S3 Bioreactor cultivation – labelling experiment 10
References 10

Overview of Tables

Table S1. LC-MS/MS gradient. 3
Table S2. MS/MS (TSQ Vantage Triple Quadrupole MS) method parameters. 3
Table S3. LC-IM-QTOFMS gradient. 3
Table S4. IM-QTOFMS (Agilent 6560) method parameters. 4
Table S5. MassHunter Quantitative Analysis QTOF CH_3-THF method parameters 4
Table S6. MassHunter Quantitative Analysis TOF CH^+=THF method parameters 4
Table S7. CDW/OD ratio of pre- & labelling experiments 10

Overview of Figures

Figure S1. 5-Methyl-tetrahydrofolic acid fragmentation proposed by Mass Frontier 5
Figure S2. Results from single standard stability test of tetrahydrofolate vitamers 6
Figure S3. LC-MS/MS analysis of 50 mg DasKO strain reconstituted in 625 µL. 7
Figure S4. Results of LC-MS/MS measurements with different quenched biomass amounts 8
Figure S5. IM separation of CH_3-THF and CH^+=THF. 8
Figure S6: Extended LC-IM-QTOFMS results for isotopologue distribution analysis 9
Figure S7. Bioreactor labelling experiment – C1 carbon source consumption & secretion 10

S1 Detailed analytical method information

S1.1. LC-MS/MS

Table S1. LC-MS/MS gradient. A = 0.1% v/v formic acid; B = acetonitrile. Flow rate was 300 µL min^{-1}.

Time [min]	% A	% B
0.00	95	5
0.50	95	5
6.50	5	95
6.51	95	5
10.00	95	5

Table S2. MS/MS (TSQ Vantage Triple Quadrupole MS) method parameters.

**Injection volume**	5 µL
**Column oven temperature**	40 °C
**Cycle time**	0.5 s
**Trigger**	10^5
**Q1&Q3 peak width (FWHM)**	0.7
**Collision gas pressure**	1.5 mTorr (~2,0 × 10^{-6} bar)
**Collision gas**	Argon
**Ion source**	Heated ESI
**Vaporizer temperature**	350 °C
**Capillary temperature**	350 °C
**Sprayer voltage**	3300
**Aux gas pressure**	15 arb
**Sheath gas pressure**	40 arb
**Ion sweep gas pressure**	0 arb

S1.2. LC-IM-QTOFMS

Table S3. LC-IM-QTOFMS gradient. A = 0.1% v/v formic acid; B = acetonitrile. Flow rate was 300 µL min^{-1}.

Time [min]	% A	% B
0.00	95	5
2.00	95	5
8.00	5	95
8.01	95	5
11.50	95	5

Table S4. IM-QTOFMS (Agilent 6560) method parameters.

**Injection volume**	15 µL
**Online mass calibration flow rate (for secondary sprayer)**	20 µL/min
**Column oven temperature**	40 °C
**Acquisition rate QTOF**	7 spectra/s
**Ion source**	Dual AJS ESI
**Ion source polarity**	positive
**Ion source gas temperature**	225 °C
**Ion source drying gas**	8 L/min
**Ion source nebulizer**	30 psi
**Ion source sheath gas temperature**	350°C
**Ion source sheath gas flow**	12 L/min
**Ion source capillary voltage**	3500 V
**Ion source nozzle voltage**	500 V
**Q1 peak width (FWHM)**	~4 m/z (Medium)
**Collision gas**	Nitrogen
**Collision energy**	18 V
**Acquisition rate TOF**	1000 ms/spectra
**IM transient rate**	19 IM-transients/frame
**IM max drift time**	50 ms
**IM TOF Transient Rate**	600 Transients/IM-transient
**IM trap fill time**	3200 µs
**IM trap release time**	150 µs
**IM multiplexing pulsing sequence length**	4 bit

S1.3. Isotopologue distribution data analysis

Table S5. MassHunter Quantitative Analysis QTOF CH_3-THF method parameters

**Isotopologue**	**TS**	**Precursor Ion [m/z]**	**Product Ion [m/z]**	**RT [min]**	**Left RT Delta [min]**	**Right RT Delta [min]**	**Extraction window ± [ppm]**
M+0	2	460.1939	313.1408	4.735	0.2	0.3	15
M+1	2	460.1939	314.1434	4.735	0.2	0.3	15
M+2	2	460.1939	315.1458	4.735	0.2	0.3	15
M+3	2	460.1939	316.1481	4.735	0.2	0.3	15

Table S6. MassHunter Quantitative Analysis TOF CH^+=THF method parameters

**Isotopologue**	**TS**	**Mass [m/z]**	**Criteria**	**RT [min]**	**Left RT Delta [min]**	**Right RT Delta [min]**	**Extraction window ± [ppm]**
M+0	2	456.1626	Close RT	4.781	0.2	0.3	15
M+1	2	457.1660	Close RT	4.781	0.2	0.3	15
M+2	2	458.1693	Close RT	4.781	0.2	0.3	15
M+3	2	459.1727	Close RT	4.781	0.2	0.3	15
M+4	2	460.1760	Close RT	4.781	0.2	0.3	15

S1.3.1 Assessment of data evaluation workflows

Data evaluation was initially performed using different vendor software options (i.e., MH Qual, MH Quant, IM-MS Browser) and in different formats (e.g., considering profile/centroid, area/height). The selection of the final approach was primarily based on the trueness of the result achieved for the natural distribution of tetrahydrofolate vitamers (^{nat}C parallel control experiments) compared to the calculated natural isotopologue fractions assessed via https://www.envipat.eawag.ch/ [1].

Centroid data gave consistently better trueness of the results in comparison to profile data. For QTOF analysis using MH Qual, the EIC of the most intense isotopologue of the quantifying peak was integrated, the corresponding mass spectrum extracted and centroid peak heights of the isotopologues exported for further calculations. The same approach for IM-TOF data was performed using IM-MS Browser. Bookmarks were set for chromatographic retention time window and arrival (drift) time of the measured for the CH^+=THF standard. The bookmarks were applied to all samples, and the corresponding isotopologue peak heights were exported for further calculations. Finally, the MH Quant workflows were found to provide accurate results for all datasets and were chosen for the final evaluation as it could be automated and involved less user intervention.

S2 Analytical developments for tetrahydrofolate vitamers

S2.1. Fragment structure assignment

The positional information of expected ^{13}C-labels is of major importance for isotopologue distribution analysis when using CID. Therefore, we assessed the most probable structure of the MS2 fragment from CID experiments using Mass Frontier 8.0 (Thermo Fisher Scientific).

Methyl-THF (5-CH_3-THF): $C_{20}H_{25}N_7O_6$ → $C_{15}H_{17}N_6O_2$

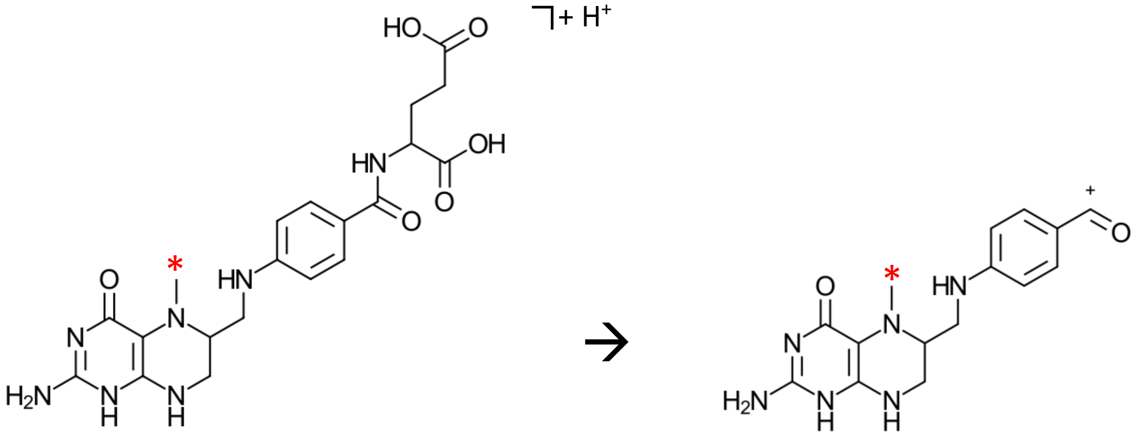

Figure S1. 5-Methyl-tetrahydrofolic acid fragmentation proposed by Mass Frontier. The expected labelled carbon (coming from formate or methanol in the THF-cycle) is indicated by an asterisk.

S2.2. Tetrahydrofolate vitamer stability and conversion analysis

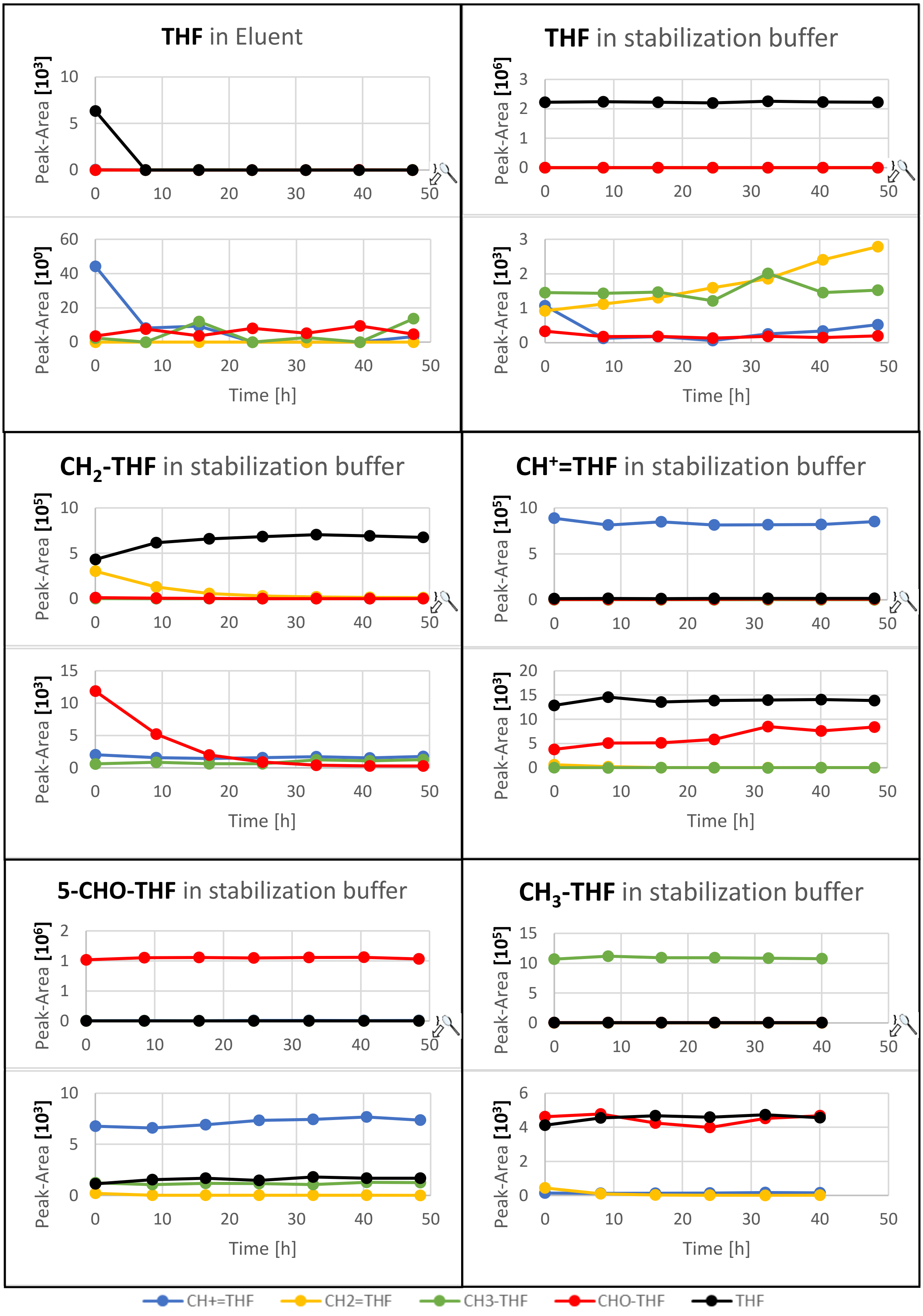

Figure S2. Results from single standard stability test of tetrahydrofolate vitamers at a concentration of 1 µmol L^{-1} at 4 °C. Lower panels are a zoom-in for all examples.

S2.3. Extraction and measurement of tetrahydrofolate vitamers of *K. phaffii*

To determine the required biomass and reconstitution volume for accurate isotopologue distribution analysis, initial experiments were performed using the established LC-MS/MS method. The DasKO strain was grown on glycerol to an OD_{600} of 33 in a shake flask. For the preculture, 10 mL YPD was inoculated with a single colony and cultivated at 25 °C at 180 rpm overnight. Subsequently, 200 mL YNB (with 10 gL^{-1} $(NH_4)_2SO_4$, 0.1 mol L^{-1} potassium-phosphate buffer, pH 6) batch culture with 18 g L^{-1} ^{nat}C-glycerol in a 2 L shake flask was inoculated to an OD_{600} of 1 (25°C, 180 rpm, 22.5 h). For metabolic sampling, four replicates of 10 mg, 25 mg and 50 mg dry cell weights (1.5 mL, 3.8 mL, and 7.6 mL cell broth, respectively) were quenched. For the reconstitution of the samples, two replicates of each biomass were reconstituted in either 625 µL or 313 µL MS-grade water. All samples were measured with the LC-MS/MS method to assess the best biomass and reconstitution volume combination. The duplicates with 50 mg quenched cells and 625 µL reconstituted dry extracts were used for LC-IM-QTOF-MS method development. Finally, to confirm that the de-polyglutamation step did not affect relative quantification, a rat serum blank was additionally assessed alongside samples. The signal intensities of folate vitamers were found to be 1000× lower than the lowest sample concentration.

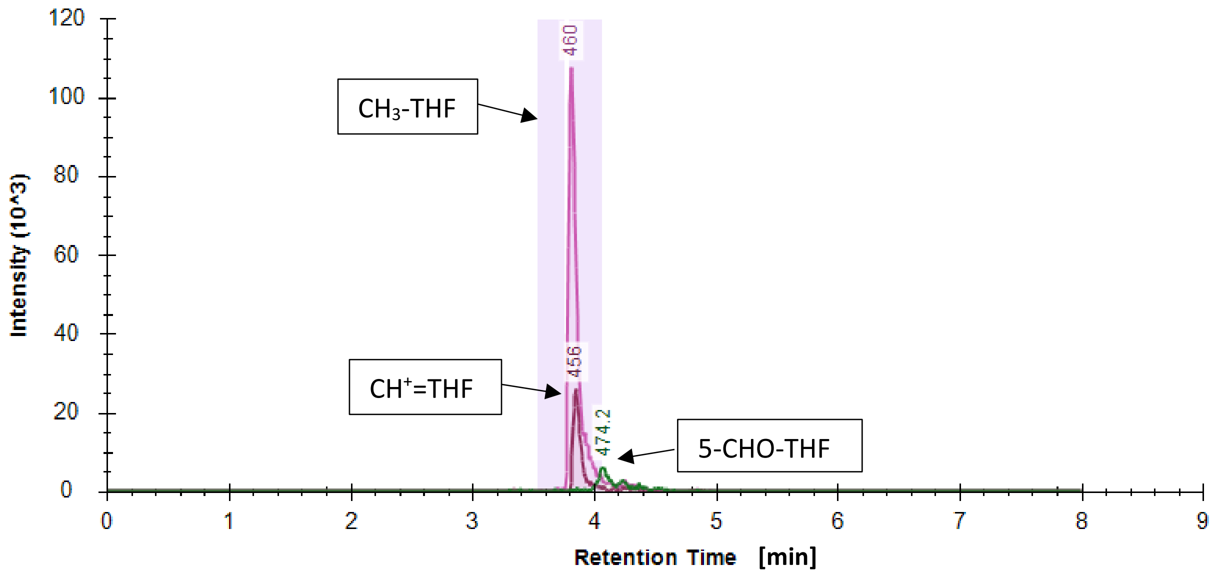

Figure S3. LC-MS/MS analysis of 50 mg DasKO strain reconstituted in 625 µL.

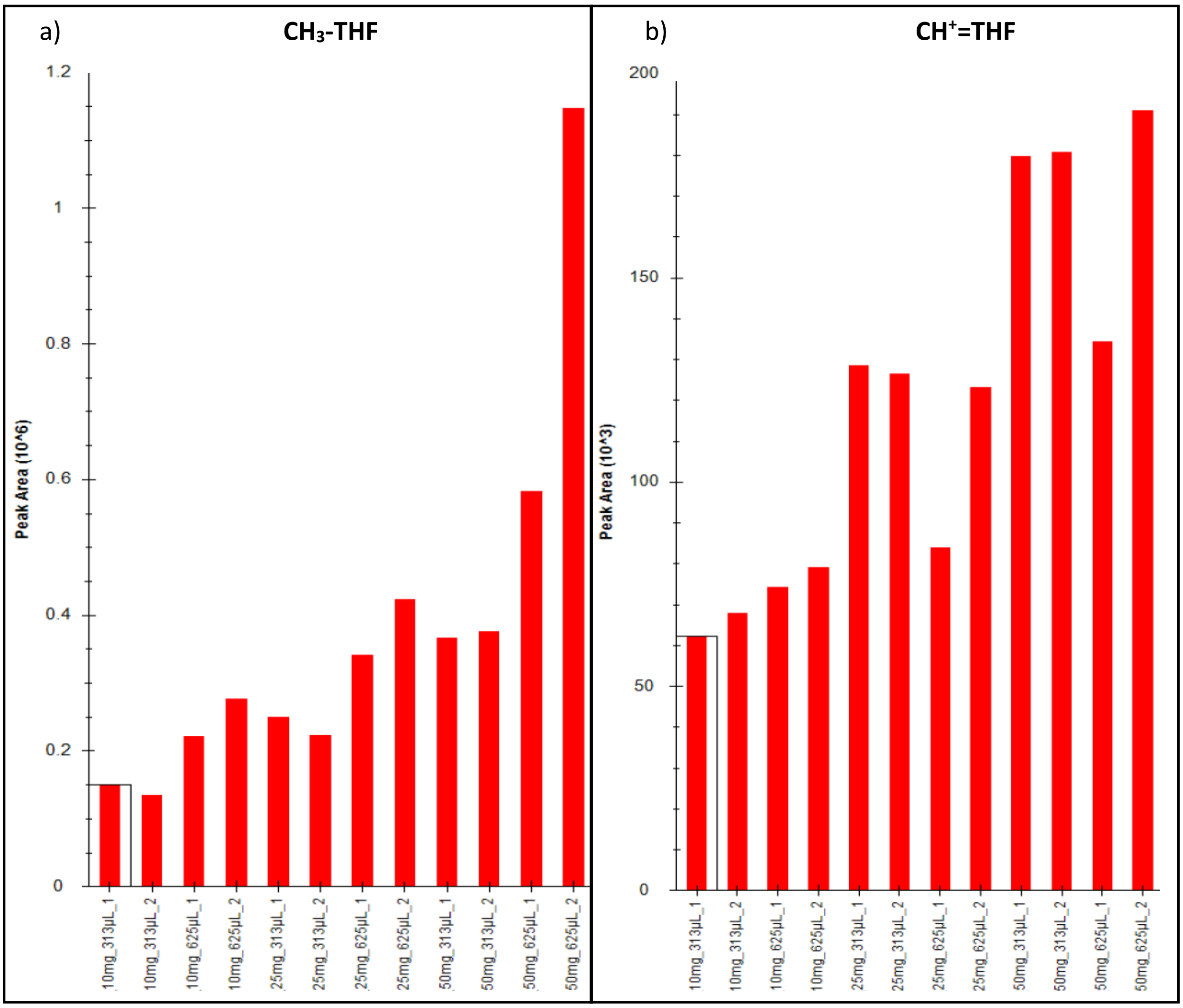

Figure S4. Results of LC-MS/MS measurements of *K. phaffii* DasKO strain cells with different quenched biomass amounts performed in duplicate. Cell dry mass was calculated via OD_{600} (10 mg, 25 mg, 50 mg) and each biomass amount was reconstituted in two different volumes (313 µL and 625 µL) to investigate matrix effects. (a) Peak areas of cell-extracted 5-methyl-tetrahydrofolate, b) peak areas of cell-extracted 5,10-methenyl-tetrahydrofolate

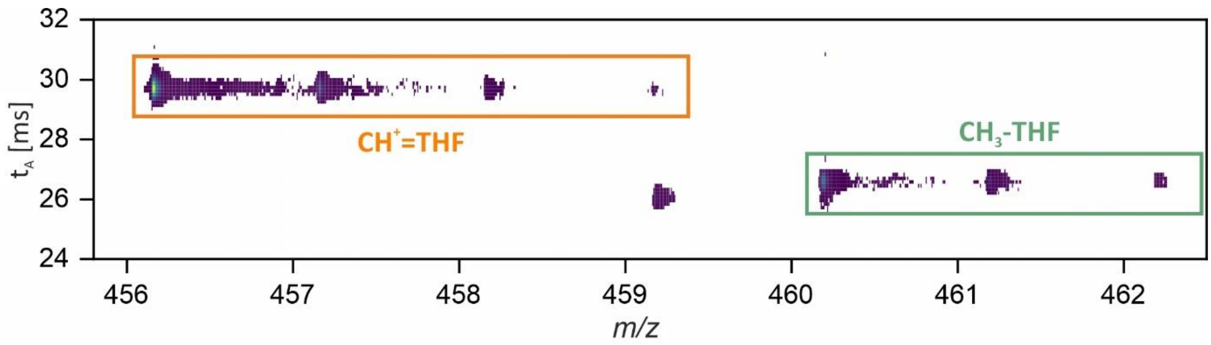

Figure S5. IM separation of CH_3-THF and CH^+=THF. Note: their chromatographic profiles are not sufficiently resolved (see **Figure S3**)

S2.3. Isotopologue distribution analysis method development

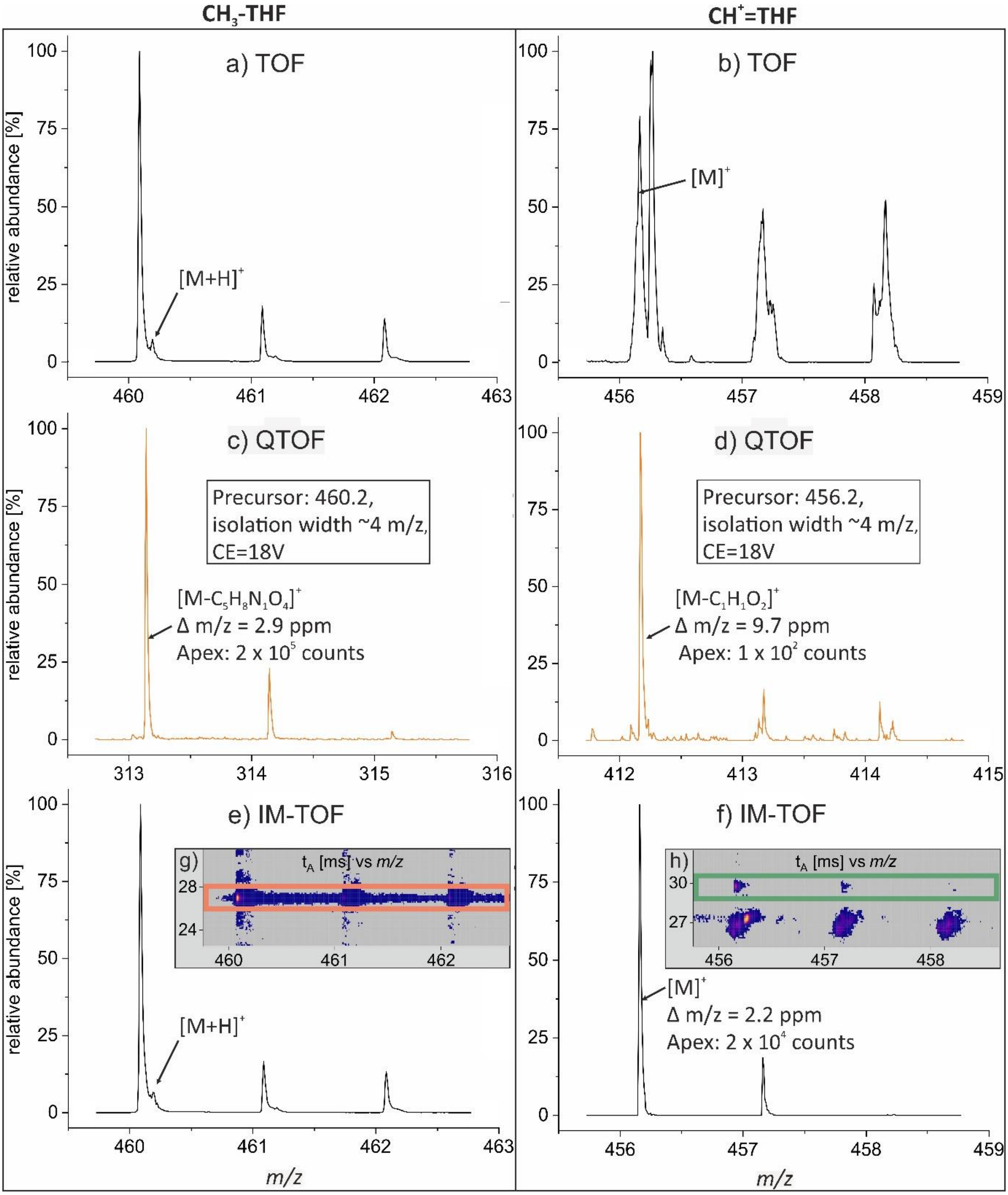

Figure S6: Extended LC-IM-QTOFMS results for isotopologue distribution analysis of folate vitamers, as a supplement for Figure 3 in the manuscript. CH_3-THF mass spectra extracted between 4.65-4.90 min retention time and 26.10-27.87 ms arrival (drift) time. CH^+=THF mass spectra extracted between 4.68-4.95 min and 29.11-30.31 ms arrival (drift) time.

S3 Bioreactor cultivation – labelling experiment

Time-resolved labelling experiments were conducted in a bioreactor with sample replicates. Due to pre-experiments, the batch end could be planned, therefore the time between batch end and induction could be limited to <1 h. Additionally, optimal carbon source usage without depletion and sample points could be planned. To optimally use 1 g ^{13}C-formate, the induction was conducted with a spike to 30 mmol L^{-1} and a second spike after 4 h. For the usage of 5 g ^{13}C-methanol, induction was conducted with a spike to 1% methanol, a second spike after 9.5 h and the last after 19 h. The C1 carbon feed and consumption/secretion profiles are shown in **Figure S7**. The CDW, OD and their ratio of the last sample points are shown in **Table S7**.

Table S7. CDW/OD ratio of pre- & labelling experiments

**Sample**				**CDW [g L^{-1}]**	**OD_{600}**	**Ratio (CDW/OD)**
WT on formate	WT on formate	8 h	^{13}C	5.224	22	0.2375
			^{nat}C	5.364	23	0.2332
	DasKO on methanol	24 h	^{13}C	5.224	22	0.2375
			^{nat}C	5.364	23	0.2332

a) b)

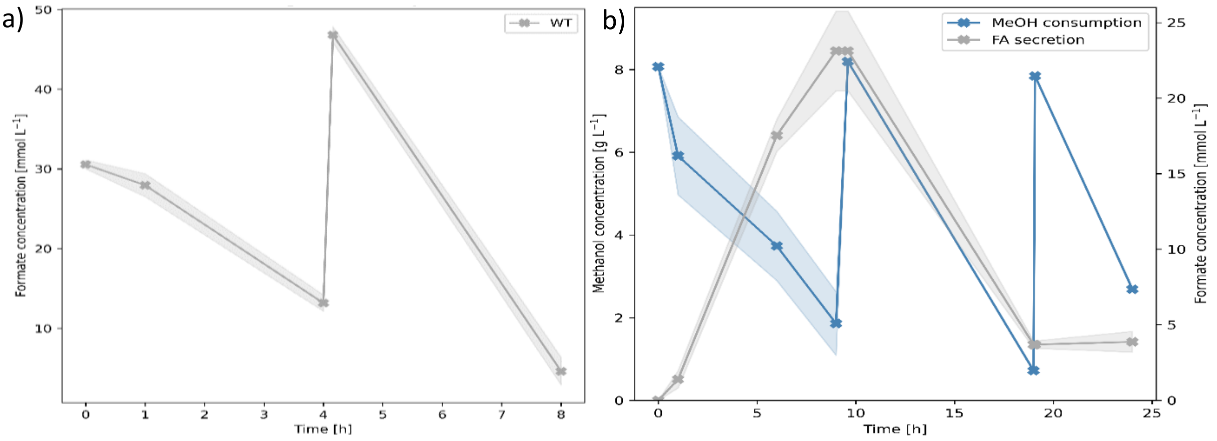

Figure S7. Bioreactor labelling experiment – C1 carbon source consumption & secretion: a) formate consumption and feeding profile, b) methanol consumption and feeding, as well as formate secretion profile

References

[1] M. Loos, C. Gerber, F. Corona, J. Hollender, and H. Singer, "Accelerated isotope fine structure calculation using pruned transition trees," *Anal. Chem.*, vol. 87, no. 11, pp. 5738–5744, Jun. 2015, doi: 10.1021/ACS.ANALCHEM.5B00941/ASSET/IMAGES/LARGE/AC-2015-00941P_0005.JPEG.
